# Effects of Constant Magnetic Field to the Proliferation Rate of Human Fibroblasts Grown onto Different Substrates: Tissue Culture Polystyrene, Polyacrylamide Hydrogel and Ferrogels γ-Fe_2_O_3_ Magnetic Nanoparticles

**DOI:** 10.3390/nano10091697

**Published:** 2020-08-28

**Authors:** Felix A. Blyakhman, Grigory Yu. Melnikov, Emilia B. Makarova, Fedor A. Fadeyev, Daiana V. Sedneva-Lugovets, Pavel A. Shabadrov, Stanislav O. Volchkov, Kamiliya R. Mekhdieva, Alexander P. Safronov, Sergio Fernández Armas, Galina V. Kurlyandskaya

**Affiliations:** 1 Institute of Natural Sciences and Mathematics, Ural Federal University, 620002 Ekaterinburg, Russia; feliks.blyakhman@urfu.ru (F.A.B.); grisha2207@list.ru (G.Y.M.); pavel.shabadrov@urfu.ru (P.A.S.); stanislav.volchkov@urfu.ru (S.O.V.); kamilia_m@mail.ru (K.R.M.); alexander.safronov@urfu.ru (A.P.S.); 2 Department of Biomedical Physics and Engineering, Ural State Medical University, 620028 Ekaterinburg, Russia; emilia1907@yandex.ru (E.B.M.); fdf79@mail.ru (F.A.F.); 3 Ural Scientific Institute of Traumatology and Orthopaedics, 620014 Ekaterinburg, Russia; 4 Institute of Medical Cell Technologies, 620026 Ekaterinburg, Russia; dyana.lougovets@mail.ru; 5 Institute of Electrophysics UB RAS, 620016 Ekaterinburg, Russia; 6 SGIKER, Universidad del País Vasco UPV/EHU, 48080 Bilbao, Spain; sergio.fernandez@ehu.eus; 7 Departamento de Electricidad y Electrónica, Universidad del País Vasco UPV/EHU, 48080 Bilbao, Spain

**Keywords:** magnetic nanoparticles, polyacrylamide hydrogels and ferrogels, cell culturing, cell proliferation, magnetic field, COMSOL modelling, magnetic properties, human dermal fibroblasts

## Abstract

The static magnetic field was shown to affect the proliferation, adhesion and differentiation of various types of cells, making it a helpful tool for regenerative medicine, though the mechanism of its impact on cells is not completely understood. In this work, we have designed and tested a magnetic system consisting of an equidistant set of the similar commercial permanent magnets (6 × 4 assay) in order to get insight on the potential of its experimental usage in the biological studies with cells culturing in a magnetic field. Human dermal fibroblasts, which are widely applied in regenerative medicine, were used for the comparative study of their proliferation rate on tissue culture polystyrene (TCPS) and on the polyacrylamide ferrogels with 0.00, 0.63 and 1.19 wt % concentrations of γ-Fe_2_O_3_ magnetic nanoparticles obtained by the well-established technique of laser target evaporation. We used either the same batch as in previously performed but different biological experiments or the same fabrication conditions for fabrication of the nanoparticles. This adds special value to the understanding of the mechanisms of nanoparticles contributions to the processes occurring in the living systems in their presence. The magnetic field increased human dermal fibroblast cell proliferation rate on TCPS, but, at the same time, it suppressed the growth of fibroblasts on blank gel and on polyacrylamide ferrogels. However, the proliferation rate of cells on ferrogels positively correlated with the concentration of nanoparticles. Such a dependence was observed both for cell proliferation without the application of the magnetic field and under the exposure to the constant magnetic field.

## 1. Introduction

Regenerative medicine focuses on the curing and replacement of whole organs or tissues damaged as a consequence of trauma, disease, age or innate defects across a wide number of dermal specific types of treatments [1,2]. One of the great problems of transplantation therapy consists in the very limited natural organ and tissues supplies. Here regenerative medicine strategies can contribute to solving this problem in some particular cases, by the implication of tissue-mimicking biomaterials.

Recently, we have proposed a new approach for cultivation of selected cell cultures, which might be useful for regenerative medicine, drug delivery and biosensor applications. This approach is based on the usage of synthetic hydrogels and ferrogels as biomimetic materials [3,4,5]. Human dermal fibroblast cultures were grown on the surface of polyacrylamide hydrogels and ferrogels [6] and their adhesive and proliferative activities were studied. Polyacrylamide hydrogels with different cross-linking density of the polymer network and polyacrylamide-based ferrogel (FG) with embedded magnetic nanoparticles (MNPs) of the iron oxide obtained by the laser target evaporation technique (LTE) [7] were synthesized and studied. Electrophysical LTE technique has very special advantage providing very large size of the batch [8] greatly requested for nanomedicine and diagnostics [9]. The obtained results were related to the heating capacity of nanoparticles [10,11], influence of the presence of LTE MNPs on the cell morphology [12], adhesion and proliferation, and therefore this research direction seems to be specially promising for cellular technologies and tissue engineering applications.

Additional value for the proposed research lines comes from the fact that today biomedical applications require very special characterization of many parameters of nanoparticles. Such a need comes from variation of the properties of nanomaterials from one to another batch to batch even for very similar fabrication conditions [12,13]. As preclinical characterization includes evaluation of many parameters, special attention is paid to manufacturing techniques offering the largest batch sizes. The above-mentioned technique of the laser target evaporation for electrophysical synthesis of spherical iron oxide MNPs provides production rates up to 50 g/h [5,7,11]. In a number of previous works with biological experiments [5,7,11,12], we used either the same batch or the same manufacturing conditions for fabrication of the LTE iron oxide nanoparticles having good biocompatibility. We believe that this adds special value to the understanding of the mechanisms of nanoparticles contributions to the processes occurring in the living systems in their presence under application external magnetic field.

The advantage of the use of ferrogel-based scaffolds for cellular technologies is an ability to operate and control the cells adhesion, proliferation and differentiation by the application of constant external magnetic field. The fundamental principles of the effect of applied magnetic field of controlled strength on living cells are not yet fully understood [14,15,16]. At the same time, they certainly should be taken into account and analyzed as complex processes involving such parameters as external magnetic field strength, magnetic field gradient and superposition of magnetic susceptibility of cell contribution and nearby environment properties. As the generation of a constant magnetic fields up to 10 kOe does not depend on complex or expensive instrumentation, it can be created with an externally located magnetic systems such as properly arranged assembly of the permanent magnets. Unfortunately, these kind of systems are still not commercially available and therefore each homemade system must be carefully characterized.

The present-day request is to study the effect of the static magnetic field of certain and well controlled strength and configuration on cells grown in vitro. In these kind of experiments both control (without external magnetic field application) and experimental (under application of a controlled magnetic field) studies must be done in the same conditions except the presence of the magnetic field of desired strength.

In general, the studies on the effect of static magnetic fields on cell proliferation are numerous, albeit their results are rather contradictive. Some researchers previously reported the positive impact of static magnetic field on cell proliferation rate. For example, 4 kOe magnetic field significantly enhanced the growth of dental pulp stem cells [17]. The effect of very weak magnetic field (10 Oe) on the same stem cells was similar [18]. Human chondrocytes growth was also promoted by 6 kOe field [19].

Some evidence showed the inhibitory effect of magnetic field on cell proliferation in vitro. The growth of human skin fibroblasts [16,20], of adipose derived stem cells (ACSs) [21] and of malignant melanoma cells [20] and cervix carcinoma lines [22] was suppressed at relatively weak magnetic field 1–5 kOe. A strong magnetic field of 70 kOe showed the same impact on melanoma, ovarian carcinoma and lymphoma lines [23].

The third group of studies of the effect of static magnetic fields demonstrates the lack of magnetic field effect on cell growth. The magnetic fields of 2–15 kOe did not cause significant differences in human fetal lung fibroblasts proliferation [24], human malignant melanoma cells. The proliferation rate of human malignant melanoma cells was also unaffected by the exposure to strong (47 kOe) field [25].

Human dermal fibroblasts (HDF) are cells responsible for synthesis of the extracellular matrix forming large proportion of the connective tissue of the skin volume. They play very important role in wound repair. HDF are considered to be an excellent model system for many aspects of cell physiology investigation. Additionally, these cells are widely used in regenerative medicine, mostly for stimulation of skin regeneration [26].

In this work, we have designed and tested a magnetic system consisting of an equidistant set of the similar commercial permanent magnets (6 × 4 assay) in order to get insight into the potential of its experimental usage in the biological studies with cells cultured in a magnetic field. To be closer to our previous findings, we used γ-Fe_2_O_3_ magnetic nanoparticles, the human dermal fibroblasts and comparatively analyzed their proliferation rate on the tissue culture polystyrene (TCPS) and on the polyacrylamide ferrogels with different concentration of maghemite LTE MNPs which physical properties were well established.

## 2. Materials and Methods 

### 2.1. Design and Characterization of Magnetic Matrix

**Figure 1:**
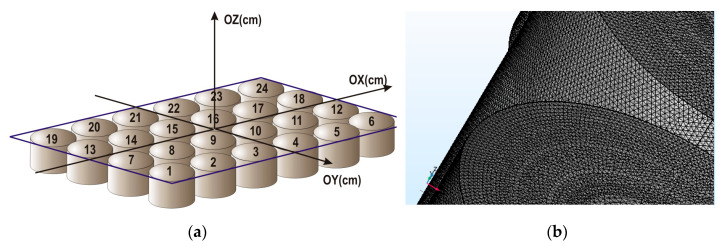
The geometry of the experiment and the model of the magnetic matrix (**a**). Segmented view of the mesh in finite-element modeling (**b**).

Commercial systems with desired magnetic field strength and spatial parameters adapted for cell culturing conditions are still not available. Therefore, the magnetic matrix was designed and tested based on 24-well polystyrene plate (Techno Plastic Products, Trasadingen, Switzerland) widely introduced for cells culturing. Such plates were used in the present study for the experiments with cell cultivation. The constant magnetic field was created by the magnetic system made by sealing the commercially available cylindrical permanent NdFeB magnets 15 mm in height and 14 mm in diameter into each well of the plate. Thus, the matrix was divided into 24 zones of 4 cm^2^, each of which included one permanent magnet (Figure 1a).

Magnetic field strength and its distribution in the magnetic system were measured experimentally by gaussmeter (State register number of approved type measuring instruments 28134-04). Magnetic measurements were carried out along three axes starting from the center of the plate: OX from −6 to +6 cm with 1 cm step; OY from −4 to +4 cm with 1 cm step and OZ from 0 to 15 cm with 1 cm step. (Figure 1a). Systematic error was evaluated in accordance with gaussemeter metrology characteristic using Equation (1) (*γ*—limits of the main relative error of measurement of magnetic induction with permanent magnetic field measurements (%), *B_L_*—limit of gaussmeter (Oe), *B_M_*—gaussmeter readings (Oe):

(1) $$\gamma =\pm \left[2.0+0.1\cdot \left(\frac{B_{L}}{B_{M}}-1\right)\right]$$

Random error was evaluated at selected points with different coordinates (X = −6, −4, 0 cm; Y = −4, −2, 0 cm; Z = 1, 2, 4, 7, 10, 13 cm) by three measurements at each point.

The degree of field heterogeneity was estimated using the root-mean-square deviation σ defined in accordance with Equation (2). An increase in the root-mean-square deviation indicates an increase in the degree of heterogeneity (*σ*—root-mean-square deviation, *H_n_*—the magnetic field value at a certain point, $\bar{H}$—the arithmetic average value of the magnetic field in the averaging area, *n*—the number of magnetic field points in the averaging area):

(2) $$\sigma =\sqrt{\frac{1}{n-1}\sum_{n}{\left(H_{n}-\bar{H}\right)}^{2}}$$

Computer modeling of the magnetic system was done as a multiphysical task that required investigation of magnetic field distribution in space. All physical and mathematical quantities of distribution under the conditions of precise topological, physical, and chemical parameters of the system were described by differential nonlinear equations in partial derivatives, which were solved by finite element method (FEM). The complexity of the FEM is that when three-dimensional system is divided into three-dimensional elements, the total number of elements became very large. It can reach 10^7^ units if it is necessary to obtain a solution with submicron resolution (for example, in zones of sharp-changing topology of curved boundaries of magnetic matrix). The construction of the FEM mesh determines the consumption of software and hardware computing resources with the necessary simulation accuracy. For this purpose, a engineering station based on the SuperMicro 4U 7047A-T (Supermicro Inc., San Jose, CA, USA) server platform with 32 3.3 GHz Intel Xeon X5 processor cores with 128 GB RAM and Comsol Multiphysics v 5.5 licensed software (license 17074991, COMSOL AB, Stockholm, Sweden, core and AC/DC module) were used.

The magnetic matrix for modeling was obtained by standard CAD drawing. It was a 6 × 4-matrix of equidistant cylinders with 14 mm diameter and 15 mm height. Hypothetical planes with cells of cultures were located parallel to the upper face of the cylinders along the *Z*-axis at the variable distances from 0 to 110 mm. Although some differences between experimental results and modelling (see below) were observed, model calculation allowed estimation of the maximum limit of the magnetic field strength and maximum uniformity zone for the geometrical parameters of the matrix and magnetic parameters of permanent magnets under consideration.

The split mesh of a non-linear stationary or temporary finite-element task is directly related to the convergence of the task. The speed of convergence and the possibility of convergence itself depends both on the solver algorithm used and on the split grid on which the solution is sought. Calculation of magnetic and electrical fields in magnetostatic conditions and surrounding areas is a complex computational task, requiring a large amount of software and hardware resources, so it is necessary to keep the number of domains broken down to a minimum. The shape and size of the finite element is determined by the topological parameters of the system, for which the changes in physical properties are possible (for example, the sharp-changing the shape of the curved boundaries of the magnetic matrix, the assumed topology of the superposition of external magnetic fields affecting cell cultures).

In this work, a tetrahedral unstructured mesh (non-uniform bonded mesh) was used, because we have a certain number of domains where the magnetic flux properties are anisotropic. The solution uses a tetrahedral mesh generator based on Delone algorithms. Figure 1b shows the partition of the magnetic matrix fragment in a plane transition in 3D dimensions. The separation was carried out by the tetrahedrons with faces 0.004 to 0.025 mm, but their spatial arrangement differed as they propagate from the axis of symmetry in the direction of increasing the z-coordinate of the plane under study. Such a grid was selected so that the magnetic flux gradient differed in different regions of cell cultures.

During the developing of the model, the following physical parameters of the materials were set. The residual magnetic induction of a single magnet, which was supposed to be used for experimental evaluation was measured using a Permagraph magnetometer (Magnetic Physik, Germany) and amounted to residual flux density M_z_ = 11.830 kG using the Z-component, which is comparable with the parameters of NdFeB permanent magnets. The magnetic permeability was μ = 1.03. The temperature considered for model calculations was 293 K. However, the results of calculations for 305 K were quite similar.

**Figure 2:**
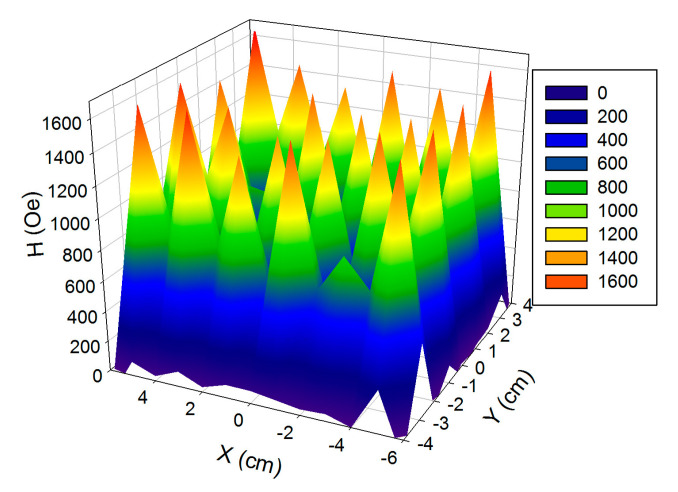
Magnetic field distribution created by the magnetic system in XY plane at Z = 0 cm.

Figure 2 shows the results of magnetic field measurements. At a distance of Z = 0 cm from the surface of the magnets, the lateral distribution of the magnetic field is non-uniform. Maximum values of magnetic field strength were observed in the centers of permanent magnets and related values exceeded the value of about 1400 Oe. The exceptions were magnets 11 and 20 where the field did not exceed 900 Oe.

**Figure 3:**
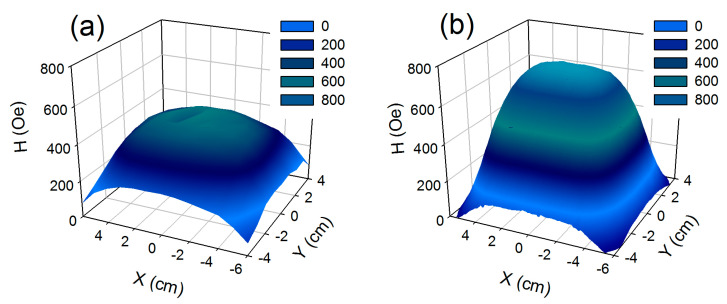
Magnetic field distribution created by the magnetic system in XY plane at Z = 2 cm: (**a**) results of measurements; (**b**) results of finite-element computer simulation.

Figure 3a shows the magnetic field distribution measured at a distance of 2 cm from the surface, of each magnet, i.e., at exact position of cell cultivation. The average magnetic field strength formed by 1–24 magnets is 380 Oe, root-mean-square deviation is 70 Oe. In the area formed by magnets (8–11, 14–17), the magnetic field strength increased to 430 Oe, root-mean-square deviation decreased to 30 Oe.

According to finite-element computer modeling (Figure 3b), the distribution of magnetic field in the magnetic system had similar shape as in the experiment. Magnetic field strength was 550 and 660 Oe, root-mean-square deviation was 140 and 60 Oe, respectively for zone (1–24) and zone (8–11, 14–17). The evaluation of the root-mean-square deviation of magnetic field strength in the model was higher than in the experiment. It might be because the model didn’t include conditions of magnetic interaction between magnets and differences between magnetic induction of each magnet. Thus, by varying the distance along the axis OZ and axis XY, it is possible to achieve the required magnetic field parameters for the designed magnetic system. In all measurements described above, the relative error did not exceed 8%.

The magnetic field created by the system of permanent magnets had maximum strength in the central area at any distance (Z) from the system. It can be explained even in frame of rather simple model of magnetic matrix as a magnetic dipole, which is located in the center of matrix (for symmetry reasons) [27]. It stems from the model that the maximum induction is observed at parallel orientation of the magnetic moment and the radius-vector. Therefore, the maximum of the intensity of magnetic field is observed near the center of XY plane.

### 2.2. Iron Oxide Nanoparticles for the Embedding in Ferrogels

Iron oxide γ-Fe_2_O_3_ magnetic nanoparticles were synthesized by laser target evaporation (LTE) method using laboratory-made equipment. In this method the iron oxide target pellet is evaporated layer-by-layer using Ytterbium fiber laser (IPG IRE-Polus, Fryazino, RF) with 1.07 µm wavelength operating in an automatic mode in pulsed regime with 5 kHz frequency. The vapors of iron oxide are then condensed in the flow of cooling gas and provide spherical MNPs. The detailed description of the LTE method and of the operating procedure is given in several previous reports [7]. Transmission electron microscopy studies were done using JEOL JEM2100 microscope (JEOL, Tokyo, Japan) operated at 200 kV.

Magnetic hysteresis loops of MNPs were measured by a superconducting quantum device (Quantum Design MPMS-7, Quantum Design Inc., San Diego, CA, USA) in the temperature range 5–30 K. Samples of about 5 mg were placed in non-magnetic capsule. Complete hysteresis loops were measured in the external field up to 65 kOe. Thermomagnetic curves (both zero-field cooled (ZFC) and field cooled (FC)) were measured for the value of external magnetic field H = 100 Oe. For obtaining zero-field cooled data, MNPs were cooled in zero magnetic field from 300 down to 5 K. Afterwards the magnetization (M) was measured with an increase of the temperature under the H = 100 Oe applied field. For FC case, H = 100 Oe magnetic field was applied both for cooling and heating of the MNPs.

### 2.3. Synthesis of Ferrogels

As-synthesized maghemite MNPs were in the air-dry state and were strongly aggregated due to the high level of their surface energy. Therefore, they could not be directly embedded into polyacrylamide matrix. Prior to the synthesis of ferrogels the electrostatically stabilized stock ferrofluid of MNPs in 5 mM sodium citrate was prepared. De-aggregation of ferrofluid was done via ultrasound treatment for 30 min using Cole-Parmer CPX-750 (Cole-Parmer, Vernon Hills, IL, USA) processor operated at 250 W. Permanent water cooling of ferrofluid was provided during the treatment. The de-aggregated ferrofluid was then centrifuged at 8000 rpm for 5 min using Hermle Z383 centrifuge (Hermle AG, Gosheim, Germany). The content of MNPs in the stock ferrofluid was 4.8% by weight.

Ferrogels were synthesized via free-radical polymerization of a monomer–acrylamide (AAm) in its 1.6 M aqueous solution at room temperature. The reaction mixture comprised the weighted amount of AAm (AAm, AppliChem, Darmstadt, Germany) and a cross-linking agent N,N′-methylene bisacrylamide (MBAA, Merck Schuchardt, Hohenbrunn, Germany) in 1/100 molar ratio to the monomer. They were dissolved in 5 mM sodium citrate for the synthesis of “blank” hydrogel, and in the mixture of stock ferrofluid with 5 mM sodium citrate (Merck Schuchardt, Hohenbrunn, Germany) for the synthesis of ferrogels. Sodium citrate and stock ferrofluid were taken in different proportion ns to synthesize ferrogels with different content of maghemite MNPs. Thus, the concentration of MNPs in the reaction mixture was 1.0 and 2.0% by weight. Polymerization of AAm was initiated by ammonium persulfate (APS) (Merck Schuchardt, Hohenbrunn, Germany), which was added to the reaction mixture in 3 mM concentration, and was accelerated by a catalyst-N,N,N′,N′-tetramethylethylenediamine (TEMED, Merck Schuchardt, Hohenbrunn, Germany) in 5 mM concentration. To meet the requirements of bio-medical testing ferrogels were synthesized in the shape of sheets between two polished glass plates (60 × 90 × 2 mm) separated by 0.8 mm spacers and sealed by silicon resin. The reaction mixture was poured into this mold by a syringe. The polymerization went on rapidly at room temperature and it took approximately 5 min for the reaction mixture to lose its fluidity. Meanwhile it was kept undisturbed for extra 60 min to complete polymerization.

After the synthesis the mold was disassembled, and gel sheets were extensively washed in distilled water for 7 days with daily water renewal to eliminate salts and unreacted monomer. During the washing water uptake of the gel sheets slightly increased due to the swelling of the gel network to equilibrium. The equilibrium water uptake of gels, in other words their swelling ratio (*α*) was determined according to the following equation:

(3) $$\alpha =\frac{m-m_{0}}{m_{0}}$$

*m*—stands for the weight of a swollen gel sample, *m*_0_—stands for the dry residue of this sample after the evaporation of all inside water in an oven at 90 °C to a constant weight.

Table 1 shows the values of the swelling ratio for all the gels used in the study. As the swelling ratio of the ferrogels was higher than water content in the reaction mixture, the resulted content of MNPs in them was lower than pre-added. It was calculated based on the composition of the reaction mixture and the equilibrium swelling ratio of ferrogels. The actual content of maghemite MNPs in ferrogels are given in Table 1.

The “blank” gel and ferrogel sheets for the biomedical studies were equilibrated for 2 days in Hanks Balanced Salt Solution (HBSS) pH = 6.8–7.2 (PanEco Ltd. Moscow, RF) with gentamicin (100 mg/L) with daily renewal and then for 2 days in 199 solution pH = 7.0–7.4, osmolality 300 ± 20 mmol/kg, buffering capacity ≤1.5 mL (PanEco Ltd. Moscow, RF) with gentamicin (100 mg/L) with daily renewal. Swelling ratio of the gels after equilibration in salt solutions is given in Table 1. After the equilibration gels scaffolds in the shape of disks 13 mm in diameter were cut off the sheets to fit the wells of the standard 24-well polystyrene plate for cell culturing. Before their use in bio-medical testing the scaffolds were sterilized in autoclave at 121 °C for 20 min.

An attempt to get direct information related to the distribution of MNPs in FG matrix was done by SEM electron microscopy using JEOL JSM-7000F scanning microscope (JEOL, Tokyo, Japan). In our earlier works [28], we proposed special protocol for SEM visualization of MNPs distribution in the sub-surface layer of composite magnetic materials comprising polymer matrix with embedded MNPs. The main shortcoming of this protocol in case of ferrogels was the preparation of the sample for the microscopy. Intact ferrogels swollen in water can not be placed into SEM column for investigation due to the inevitable water evaporation. Therefore, gel samples were completely dried out at room temperature during 48 h. In the dry state the properties of ferrogels were similar to the properties of conventional polymer composites filled with MNPs. As carbon is the most common element in polymers, we therefore deposited the carbon film of about 20 nm using sputtering technique in order to ensure high surface conductivity of the samples for SEM.

Magnetic hysteresis loops of the “blank” gels and ferrogels were measured by a vibrating sample magnetometer (Cryogenics, Ltd. VSM, London, UK) at room temperature. Samples of about 70 mg were placed in non-magnetic water-stable polymer capsule. Complete hysteresis loops were measured in the external field up to 1.8 kOe.

### 2.4. Cell Proliferation Assay

The primary dermal fibroblasts were isolated from skin samples. The samples were surgically excised from two patients (donors #1 and #2). Before the intervention, the patients have signed the written informed consent. The study was approved by the Ethics Committee of the Institute of Medical Cell Technologies, Ekaterinburg, RF. Skin samples were dissociated by collagenase I (Sigma-Aldrich, St. Louis, Missouri, USA) for 2.5 h at 37 °C, then enzyme was inactivated. Dissociated cells were washed and re-suspended in special medium. This growth medium consisted of mixture of Dulbecco modified Eagle’s medium (DMEM) and medium F-12 (1:1) (Gibco, Thermo Fisher Scientific, Waltham, MA, USA) supplemented with 12% fetal calf serum (Gibco, Thermo Fisher Scientific, Waltham, MA, USA), glutamine (0.03%) and gentamycin (50 μg/mL). Cells were grown in culture flasks (Nunc, Roskilde, Denmark) in CO_2_-incubator MCO-15AC (Sanyo/Panasonic, Moriguchi, Osaka, Japan) at 37 °C, 5% CO_2_ and 100% humidity. Fibroblasts were subcultured when the cell monolayer reached 80% confluency. Cells were detached from flask surface by 0.25% trypsin with ethylenediaminetetraacetic acid (EDTA, Gibco, Thermo Fisher Scientific, Inc., Waltham, MA, USA). The quantity of viable cells was measured after staining with trypan blue by cell counter TC-20 (Bio-rad, Hercules, CA, USA).

Fibroblasts from passage 5 were used in experiment. After trypsinization cells were suspended in growth medium, the mixture was poured in wells of 24-well TCPS plates (Techno Plastic Products, Trasadingen, Switzerland). Cell viability in suspension was ≥95%, the seeding density was 3000 viable cells/cm2 for all types of substrates in all experiments. Plates with cells were incubated in CO_2_-incubator for 4 days being exposed (or without exposure in control) to the magnetic field. After incubation cells were washed with phosphate buffer solution, fixed with 2.5% glutaric aldehyde for 24 h at 4 °C and then dehydrated by sequential exposure to ethanol solutions with increasing concentration (30, 50, 70 and 95%) for 10 min. for each concentration. The cytoplasm of fixed cells was stained with 0.3% pyrazolone yellow solution, cell nuclei were stained with 4′,6-diamidino-2-phenylindole (DAPI, Sigma-Aldrich, St. Louis, MO, USA).

Cells were visualized using fluorescent microscope Axio Lab A1 FL (Carl Zeiss, Oberkochen, Germany) at ×100 magnification in FITC (for pyrazolone yellow) and DAPI channels. For every well, nine fields of view were captured and analyzed. The area of the field of view was 0.93 mm^2^. For image analysis, the ImageJ software (Wayne Rasband, NIH, Bethesda, MD, USA) was used. The quantity of cells was calculated by the number of their nuclei.

### 2.5. Design of Experiments with Cell Cultures

**Figure 4:**
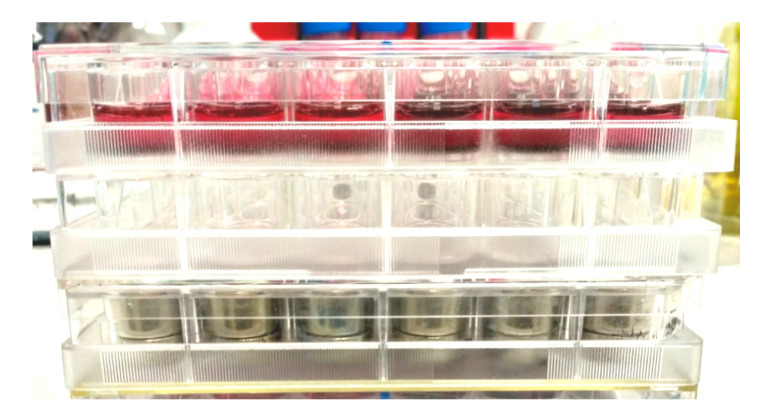
Arrangement of the cellular experiments with the use of plates’ stack. See explanation in text.

For the evaluation of the influence of magnetic field on proliferation of fibroblasts the stack of three identical plates was installed (Figure 4). The lower plate was the calibrated magnetic matrix (see Section 2.1) producing the magnetic field of defined strength. Additional empty plate and the experimental plate with cells were placed above the magnetic matrix. Such configuration provided the distance about 20 mm between the surface of magnets and the bottom of experimental plate with cells. The stack was placed on the lower shelf of CO_2_-incubator, the other 24-well plate with cells (control) was placed on the lower shelf of incubator at a distance about 35 cm from the upper shelf. At this distance, the external magnetic field strength was below 5 Oe (using the exponential approximation of the experimental data) even in a central part of the control cultural plate. Besides, the control plate was protected from magnetic field by iron sheet. After cultivation in CO_2_-incubator for 4 days cells were fixed, and the density of cell monolayer was estimated.

The cell monolayer density was presented in the form *X* ± *m*, where *m* was the standard error of the arithmetic average *X* (the quantity of cells per cm^2^). In some cases, the mean number of cell population doublings (*PD*) was calculated as: *PD = log_2_ × N/N*_0_, where *N* is the cell monolayer density after cultivation; *N_0_* is the seeding density. The non-parametric Mann–Whitney U-test was used to compare statistical significance of the difference between two independent groups with a level of significance set at 0.05. Statistical data processing was performed using the application software package “STATISTICA 6.0” (Statsoft, Dell, Round Rock, TX, USA).

## 3. Results

### 3.1. Properties of Nanoparticles and Ferrogels

**Figure 5:**
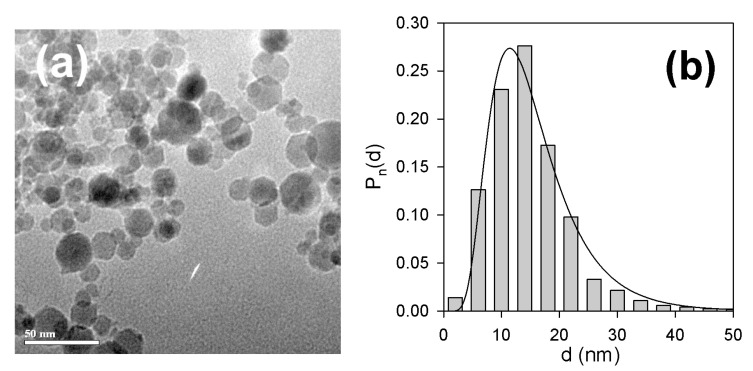
(**a**) TEM image (JEOL JEM2100) of iron oxide magnetic nanoparticles (MNPs) used in the synthesis of ferrogels; (**b**) particle size distribution obtained by the graphical processing of 2570 images. The line corresponds to the lognormal fitting of the histogram.

**Figure 6:**
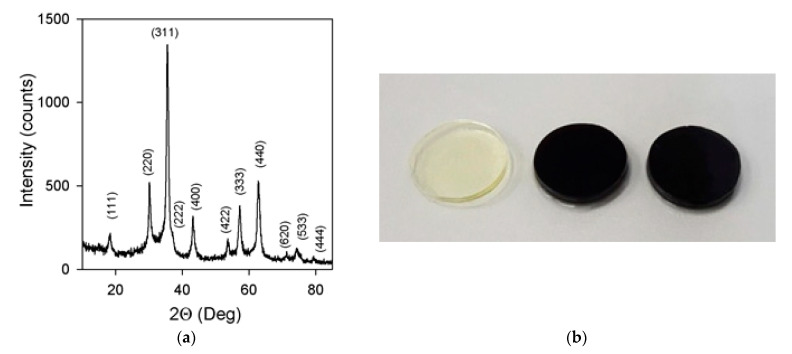
(**a**) XRD pattern of iron oxide MNPs with Miller indexes for the inverse spinel lattice of magnetite (or maghemite). (**b**) Gel samples used for cells culturing (from left to right): BG, FG1, FG2. Diameter of gel samples is 13 mm.

Figure 5a shows typical transmission electron microscopy (TEM) image of iron oxide MNPs. Most of the particles were spherical; some of them had a hexagonal shape. Their typical diameter was 5–30 nm but a small number of larger particles (Figure 5b) was also detected. The particle size distribution (PSD) given in Figure 6b was a result of graphical processing of 2570 images. PSD histogram was fitted well by lognormal probability function:

(4) $$P_{n}(d)=\frac{3.467}{d}\exp\left[-\frac{{\left(\ln d-\ln d_{0}\right)}^{2}}{2\sigma^{2}}\right]$$

with a median *d*_0_ = 14.1 nm and a standard deviation for the natural logarithm of diameter *σ* = 0.462. The number-average and volume-average diameters calculated from PSD were *d_n_* = 14.9 nm and *d_w_* = 28.3 nm respectively.

The specific surface area (*S_sp_*) of MNPs was measured via low temperature adsorption of nitrogen by Brunauer–Emett–Teller calculation procedure using Micromeritics TriStar3000 analyzer (Micromeritics, Norcross, GA, USA). It was found 78.1 m^2^/g. In case of spherical particles, the specific surface area can be used for the estimation of mean diameter of particles according to the following equation [29]:

(5) $$d_{BET}=\frac{6}{\rho S_{sp}}$$

In this equation *ρ* stands for the density of iron oxide MNPs. Using the value *ρ* = 4.6 g/cm^3^ the estimation of mean particle diameter gave *d_BET_* = 16.7 nm, which was fairly close to *d_n_* value, calculated from PSD.

Phase composition of MNPs was examined using X-ray diffractometer Bruker D8 Discover (Bruker Corporation, Billerica, MA, USA) operated at Cu K_α_ radiation (wavelength λ = 1.5418 A) with a graphite monochromator and a scintillation detector. Corresponding diffractogram is given in Figure 6a. It was processed using built-in Bruker software TOPAS-3 provided Rietveld full-profile refinement. The crystal structure corresponded to an inverse spinel cubic lattice with space group Fd3m, which is characteristic both for magnetite (Fe_3_O_4_) and maghemite (γ-Fe_2_O_3_).

In fact, the inverse spinel cubic lattice covers the variety of non-stoichiometric iron oxides, which differ in the number of vacancies in the Fe sub-lattice (hence in Fe oxidation degree), starting with magnetite with the lowest oxidation degree and ending with maghemite with the highest oxidation degree [27]. The actual non-stoichiometry of MNPs was determined by Red/Ox potentiometric titration by potassium dichromate using automatic titrator Schott Titroline (Schott AG, Mainz, Germany). The actual chemical composition of MNPs was found Fe_2.02_O_3_, which was very close to maghemite (γ-Fe_2_O_3_).

The hydrodynamic diameter of particles/aggregates in ferrofluid was measured by the dynamic light scattering (DLS) using Brookhaven ZetaPlus analyzer (Brookhaven Instruments, Holtsville, NY, USA). It was found equal to 46.6 nm. It is known that the value of hydrodynamic diameter in polydisperse suspension corresponds to the 5th momentum of PSD [30], which is also called the “intensity-average” diameter. The value of the “intensity average” diameter calculated from PSD (Equation 5) was 40.3 nm, which is very close to the hydrodynamic diameter, determined via DLS. Based on these data we have made a reasonable assumption that the stock ferrofluid comprised individual maghemite MNPs.

**Figure 7:**
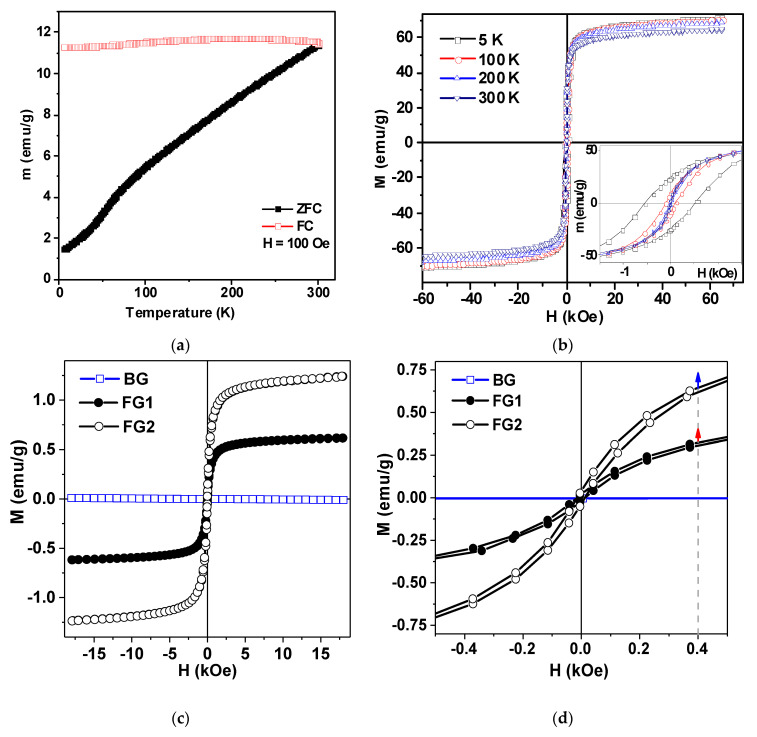
(**a**) Zero-field cooled and field cooled (ZFC-FC) thermomagnetic curves of air-dry laser target evaporation (LTE) iron oxide MNPs; (**b**) magnetic hysteresis loops of LTE iron oxide MNPs measured for fixed selected temperatures; (**c**) magnetic hysteresis loops of blank gel (BG) and ferrogels FG1 and FG2; (**d**) magnetic hysteresis loops of blank gel and ferrogels in the low-fields region. Dashed line indicates external magnetic field strength of 400 Oe, i.e., typical field strength created by magnetic system in the experiments with cell cultures. Blue arrow indicates maximum value of saturation magnetization for FG2 and red arrow indicates maximum value of saturation magnetization for FG1.

Figure 7a shows ZFC-FC curves and hysteresis loops for maghemite MNPs. They were measured by a vibrating sample magnetometer in the temperature range 5–30 K. Analysis of the shape of the hysteresis loops measured at different temperatures shows that full saturation was not reached even in the field of 65 kOe. We therefore used the magnetization value for this field as the saturation magnetization value (M_s_) just for simplicity. The M_s_ ≈ 62 emu/g is reasonable for maghemite MNPs of about 17 nm in diameter [7,31]. Core-shell magnetic model without consideration of inter particle coupling was previously proposed and tested. In this model each MNP was described having two parts: a ferrimagnetic core and a surface shell of thickness delta, where the spins were frozen with no long-range magnetic order [7]. The magnetic response peculiarities of such a material was attributed to the large random exchange fields and the concomitant anisotropy, corresponding to the core coupled to the spins of the surface. The magnetization behavior when approaching saturation and non-zero but quite low coercivity at room temperature are consistent with the core-shell magnetic model proposed for maghemite MNPs. However, one additional comment should be made here. Although, Ms value close to 62 emu/g is consistent with existing reference data [7] this value corresponds to the highest level reported in the literature. One should realize that available data from different sources are not easy to compare because in all cases MNPs have particular size distributions even for close average size numbers. One of the possible reasons for observed results is the presence of some amount of MNPs (Figure 7b) with higher individual M_s_ values. Obtained M_s_ value is just about 20% below the M_s_ value for a bulk maghemite. We will use this number below for the analysis of magnetic properties of ferrogels.

Figure 7c,d show magnetic hysteresis loops of gel and ferrogels. Ferrogels were filled with maghemite LTE MNPs: concentration in ferrogel FG1 was 0.63 wt % and in FG2 it was 1.19%. As expected, clear diamagnetic response (due to the high water content in the gel) was obtained for blank gel and an “S” type shape of M(H) loop was evident for the ferrogel. Subtraction of the blank gel signal from the signal of ferrogel confirmed that the indicated concentrations of the MNPs were correctly insured during synthesis. Obtained values of magnetization for gel and ferrogels can be useful for practical evaluation of functional properties of these materials. For example, one can clearly define magnetic moment corresponding to each ferrogel in the magnetic field H = 400 Oe, corresponding to the field of biological experiments with designed magnetic matrix. We can also define the up limit for magnetization of each material using earlier mentioned magnetization limit of 20% for bulk maghemite. Although such a procedure gives just a rough approximation, application of 20% increase rate (Figure 7d) shown by arrows gives clear evidence of the order of magnitude for M values.

An important question concerning the inner structure of ferrogels is the distribution of maghemite MNPs in polyacrylamide (PAAm) matrix. As it was mentioned above there is a strong evidence that the precursor ferrofluid used for the synthesis of ferrogels comprised statistically dispersed individual MNPs. There were no signs of MNPs aggregation in the reaction mixture like turbidity or opalescence. The polymerization was rapid and there were no such signs in the resulted ferrogels also. So, it was reasonable to assume that the distribution of MNPs in ferrogels remained basically the same as in the precursor ferrofluid.

Up to now, there is no reliable experimental technique to observe distribution of MNPs in gel matrix directly. In the case of gel or ferrogel, the majority of available estimation are given either by investigation of dried or freeze-dried samples [32]. Of course, the most important question to what extent we can recover the main structural features of a swollen intact ferrogel based on these data.

**Figure 8:**
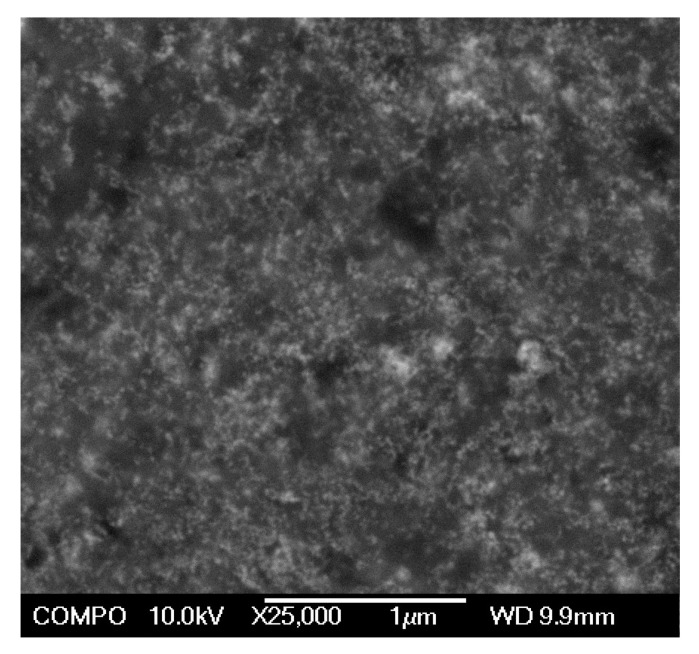
SEM image of dried FG1 sample (7.7 wt. % of MNPs in dried state, 0.63 wt. % in swollen state) covered with 20 nm conductive carbon layer in order to enhance conductivity. Brighter agglomerates remind the aggregations of MNPs.

Figure 8 gives a typical SEM image of dried ferrogel. The MNPs in its structure are noticeable as unevenly shaped light spots. They are more or less uniformly distributed over the bulk of dry gel matrix. Certain aggregation of MNPs might as well be noticed. However, it is hard to tell whether is occurs in the swollen gel, or it is the result of ferrogel shrinkage during drying. The extent to which the intact structure of the swollen ferrogel is changed might be estimated based on the swelling ratio values given in Table 1. These values are in fact equal to the amount of water, which evaporates during drying. As the samples lost approximately 12–13 volumes of water in this procedure (see Table 1), their dimensions were reduced approximately by the cube root, i.e., by the factor of 2.3–2.4. Thus, one may assume in zeroth approximation that the inner structure of ferrogels might be like presented in Figure 8 with all distances enlarged by about 2.4 times.

There should be considered an option for MNPs embedded in a ferrogel to leave it during its swelling and storage in water. In principle, this opportunity takes place if the diameter of MNPs is smaller than the characteristic mesh size of a polymeric network. The mesh size of gels depends on the concentration of a monomer and a cross-linker in the reaction mixture used in synthesis. The composition of the reaction mixture in the present study was the same as that in ref. [5]. The mesh size of the resulted ferrogels in this case was evaluated as 2.4 nm [5]. This value is substantially lower than the value of the diameter of MNPs at any mode of averaging (*d_n_* = 14.9 nm, *d_w_* = 28.3 nm, *d_BET_* = 16.7 nm, see above). It means that the MNPs are effectively entrapped in the PAAm network and can not leave it. The visual observation and analytical testing confirmed this conclusion. No signs of MNPs exudation during washing of ferrogels was observed. The analysis by spectrophotometry showed that the total concentration of iron in any forms in the supernatant water in contact with ferrogels was 0.3 mg/L, which is the typical level of the concentration of iron ions provided by the surface dissolution of iron oxides in water at ambient conditions [33].

### 3.2. Cells Proliferation on TCPS and Ferrogells in a Magnetic Field

In the first experiment the growth rates of fibroblasts on the surface of standard 24-well plates made of tissue culture polystyrene (TCPS) with and without application of the external magnetic field were compared. The purpose of this experiment was to study the possible effect of magnetic field and the contribution of its heterogeneity on cell proliferation in the case of tissue culture polystyrene substrate.

**Figure 9:**
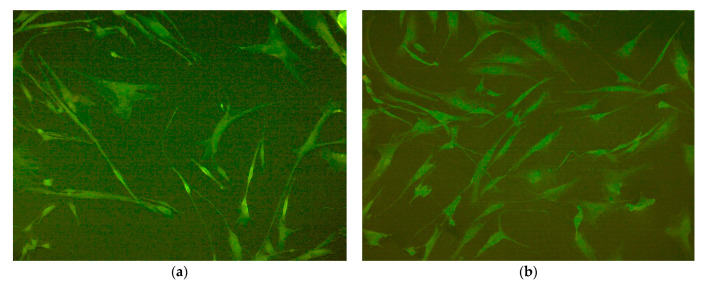
Human dermal fibroblasts on tissue culture polystyrene (TCPS) without magnetic field application (**a**) and in magnetic field (**b**) after 4 days of growth. Magnification ×100, staining of the cell cytoplasm with pyrazolone yellow.

Fibroblasts obtained from donor #1 were used in this experiment. After 4 days of cultivation, the majority of the fibroblasts grown in magnetic field and the fibroblasts grown in its absence had typical spindle-shaped morphology with several long processes (Figure 9). Cells sizes ranged from 100 to 300 microns in length and form, 10 to 50 microns in width, and they strongly depended on the degree of cell spreading. The density of cells grown in an external magnetic field was noticeably higher than in the control case. The photos were taken from wells #9 of both plates for correct comparison (see also Figure 1).

The cell monolayer densities data for the central and for the peripheral plate wells both in magnetic field and in control state are shown in Table 2. One can see that the cell density was higher (20 ± 5% in comparison with control) in wells exposed to magnetic field regardless of their central or peripheral location. Besides, the monolayer density was approximately the same in the central and in the peripheral wells for both magnetic field and control. Therefore, the heterogeneity of magnetic field in central and peripheral parts of magnetic matrix did not have an effect on cell proliferation.

In the second experiment fibroblasts obtained from donor #2 were used. Cells were seeded on PAAm blank gel (BG) and ferrogels (FG1, FG2), which composition is given in Table 1, and on the TCPS. Seeding on each type of substrate was replicated in six wells for both plate in magnetic field and control plate (unexposed to magnets) using the scheme presented in Figure 1a. According to this scheme, wells with each type of substrate were selected symmetrical to the center of magnetic matrix. It allowed to equalize the magnetic field distribution for each group of wells, and hence, to neutralize the possible influence of heterogeneity of magnetic field on the results of experiment. As in the previous experiment, cells were grown during 4 days in CO_2_-incubator.

**Figure 10:**
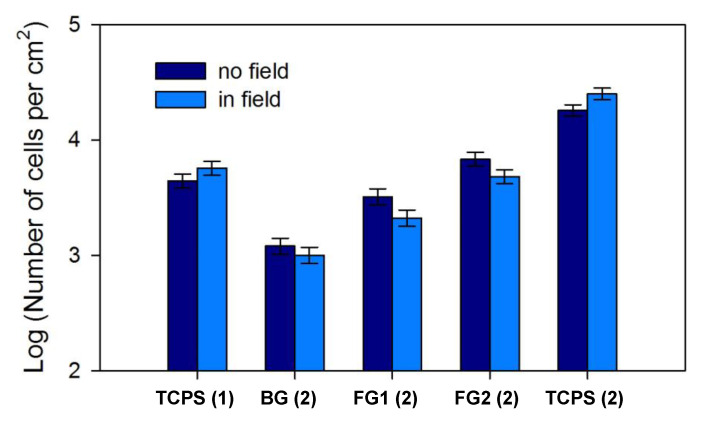
The effects of a magnetic field on the fibroblasts’ proliferation rate in different scaffolds. Data are presented as *X ± m*, n = 54 (9 fields of view per well ×6 wells). Label TCPS (1) corresponds to the proliferation of fibroblasts from donor #1, the numerical values are given in Table 2 (all 24 wells). All other labels correspond to the proliferation of fibroblasts from donor #2. A level of significance *p* < 0.05 was determined for FG1 (2), FG2 (2), and TCPS (2).

The results of the experiment demonstrated the significant effect of magnetic field and MNPs concentration on fibroblasts growth (Figure 10). Donor #2 fibroblasts had much higher proliferation rate on plastic, than donor #1 fibroblasts from the previous experiment, which is, obviously, due to individual features of these strains. On the plastic surface fibroblasts exposed to magnetic field formed the monolayers with statistically higher density, than unexposed control cells, which was in agreement with the results of previous experiment.

The effect of magnetic field on the growth of cells on PAAm blank gel (BG) and PAAm ferrogels (FG1, FG2) was opposite to that for the cells on TCPS: the magnetic field suppressed the proliferation of fibroblasts on ferrogels (Figure 10). At the same time, the proliferation rate of cells on ferrogels positively correlated with the concentration of MNPs (differences between cell monolayer densities on gels with various concentrations of MNPs were statistically significant in all the cases). Such a dependence was observed both for cell proliferation without the application of the magnetic field and under the exposure to the magnetic field.

In Table 3, the results of the experiment on the cell proliferation on gels and TCPS are presented in the form of the mean number of cell population doublings. The data using this parameter demonstrate the same trends that were already shown using absolute cell density values: almost complete absence of fibroblasts proliferation on blank gel, the direct dependence of the number of cell population doublings from the concentration of MNPs in gel and the decrease of cell proliferation rate on gels exposed to static magnetic field.

## 4. Discussion

The mechanism of the biological activity of static magnetic field on cells is still not clear. It is often assumed that external field affects the concentration and activity of free radicals such as reactive forms of oxygen (ROS). In some studies, the increase of ROS levels in cells after exposure to the magnetic field was detected, which can cause oxidative damage on cell structures and impair metabolic processes [20]. Alternatively, other researchers reported on the absence of effects on reactive forms of oxygen levels in cells exposed to magnetic field [34]. It should be noted, that the ROS effect on cells may not only be harmful, but at moderate concentrations can also positively regulate cells functions [35].

The cell proliferation rate can also be affected by the magnetic field-induced activation of Ca^2+^ channels. According to Lew et al. hypothesis [17], the plasma membrane structure is affected by a magnetic field, which causes the modification of ion channels followed by Ca^2+^ ions influx. The intracellular calcium triggers the cytoskeleton reorganization, which influences cell proliferation. Thus, the application of an external magnetic field definitely affects cells through various mechanisms with different impact on their proliferation. The effect of external field on cells replication may vary greatly depending of cell type, field strength, and other experimental conditions, that can explain the discrepancy in results obtained by researchers. Possibly, the above-mentioned mechanisms can be responsible for the fibroblasts proliferation rate in magnetic field of 400 Oe that was applied in the present study. The most surprising result we have obtained is the opposite influence of the magnetic field of particular characteristics on the proliferation of cells cultured on the polystyrene and ferrogels (see Figure 10). Unexpectedly, the magnetic field seems to impair the proliferation activity of cells on ferrogels with MNPs, while increasing it if applied in the course of grows on the TCPS.

First, let us focus on the adhesion and the proliferation of cells in the absence of the external magnetic field. The proliferation rate clearly depends on the nature of the scaffolds used. The highest rate was observed on TCPS of commercial plates for cells culturing. The proliferation rate on PAAm gel and PAAm ferrogels was lower. To some extent, it is due to the difference in the chemical nature of these types of surface. However, other factors can also make important contributions. The surface of polystyrene plate is specially treated by plasma discharge to provide partial oxidation of polystyrene chains and to modify the surface with carboxyl residues [36]. Carboxyl residues can take part in H-bonding with proteins, that potentially enhance adhesion of cells and favors proliferation. In this respect, the surface of PAAm gel and PAAm ferrogels contains amide residues, which are not eager to form H-bonds unlike carboxyls. Therefore, the proliferation rate at PAAm hydrogel is substantially lower that that at TCPS. Meanwhile, the switch from PAAm blank gel to PAAm ferrogel improves proliferation. It might be a consequence of the fine surface structure of ferrogel. This structure might be different from the surface of PAAm blank gel due to the loss of close to surface MNPs and formation of extra fine pores. Unfortunately, the described above technique of evaluation of the surface properties of gel and ferrogel is inappropriate for fine analysis. Actually, there are no appropriate experimental techniques for such kind of studies.

The chemical nature of the surface of PAAm ferrogel is basically the same as for the surface of PAAm gel itself. At the same time, it is clearly noticeable that the proliferation substantially increases if MNPs are embedded into PAAm gel matrix (see Figure 10). This result is in good agreement with our previous observations [5,6,37]. However, the underlying reason for such enhancement of proliferation rate by MNPs is not clear yet. One of the possible factors is the negative electrical potential of the ferrogel. It was shown earlier [5,37] that the electrical potential of PAAm ferrogel with embedded iron oxide MNPs is around minus 30–40 mV. The electrical potential of a ferrogel originates from the ferrofluid, which is the precursor in the synthesis of ferrogel. The precursor ferrofluid is electrostaticlly stabilized by sodium citrate. Citrate anions are adsorbed on the surface of MNPs and provide their net negative electrical charge. Negatively charged MNPs repel each other and that prevents aggregation and sedimentation. Being embedded in ferrogel, MNPs preserve their negative electrical charge. Meanwhile, unlike ferrofluid, MNPs in ferrogel are immobilized and can not leave the gel network. If the electrically charged species are not able to move across the boundary of the gel, it establishes the Donnan equilibrium at the boundary and the resulting Donnan electrical potential [5,37,38]. The higher the content of the MNPs, the higher in the Donnan potential. In general, the electrical potential of the surface is a favorable factor for the adhesion of cells.

Next possible favorable factors of the increase of cells proliferation rate in ferrogels are the gel rigidity and/or the gel roughness (porosity) change with the growth of MNPs concentration in PAAm gel. It is well known that both of these factors are strong determinants of the scaffold biocompatibility [36,39,40,41,42,43]. Previously, we have shown that the addition of MNPs to the PAAm gel network results in significant increase of the Young’s modulus [5,37] and contributes to the surface roughness [6] of the PAAm composite material. In this work, we also presented indirect evidence of the existence of MNPs and their aggregates at the surface of ferrogels (see Figure 8). Thus, at least three major factors, and in particular an increase in charge and stiffness and surface roughness of the PAAm gel, can take part in the positive impact of MNPs on the biocompatibility of ferrogels.

Let us consider now the influence of the magnetic field on the cell proliferation rate that we obtained in all series of experiments with the fibroblasts used. Whichever mechanism of the influence of the magnetic field could be, no doubt it should be very complicated. From this point of view, it would not be surprising if the influence of the magnetic field presents itself in the alternative ways at different substrates. While on TCPS we observed the enhancing effect of the magnetic field and the proliferation rate of cells on PAAm gel and PAAm ferrogels we observed suppression of the proliferation. Considering the differences in chemical structure and surface charge between TCPS and PAAm gel, the mechanisms of the adhesion of cells on these substrates can be rather different. The variabilities in cell adhesion mechanism can result in different impact of the magnetic field on the proliferation of cells.

At the same time, the negative impact of magnetic field on the proliferation rate of fibroblasts is also different on PAAm gel and PAAm ferrogels (see Figure 10). On ferrogels, the magnetic field effect is much more pronounced. This result itself implies a role of MNPs in the determining of cell proliferation on ferrogels at the presence of magnetic field. Therefore, we may assume the indirect influence of magnetic field on the cell interaction with ferrogel-based scaffolds. In other words, the driven force of the magnetic field is able to change some physical properties of ferrogels that determine the cell adhesion and proliferation: charge, rigidity/stiffness and surface roughness, or ferrogel porosity. The search for the most important determinant of these three is the issue for our future researches.

## 5. Conclusions

In this work, the magnetic matrix was designed based on a 24-well polystyrene plate for the experiments with cell cultivation. The magnetic system was made by sealing the commercially available cylindrical permanent magnets into each well of the plate. Magnetic field distribution in the magnetic system was carefully measured experimentally and modelled using FEM. The distribution of magnetic field in the magnetic system had similar shape according to the experimental and model evaluations. Iron oxide γ-Fe_2_O_3_ magnetic nanoparticles were synthesized by laser target evaporation method using the same synthesis parameters as in our previous studies. Ferrogels were synthesized via free-radical polymerization of a monomer–acrylamide in aqueous solution at room temperature. The concentration of MNPs in ferrogel was 0.63 and 1.19% by weight.

Human dermal fibroblasts were used for the comparative study of proliferation rate on polystyrene, polyacrylamide blank gel and ferrogels. The opposite effect of magnetic field on the growth of cells on PAA gels and on TCPS was observed. The application of the magnetic field of the order of 500 Oe suppressed the proliferation of fibroblasts on ferrogels, but stimulated it on TCPS. However, the proliferation rate of cells on ferrogels positively correlated with the concentration of iron oxide MNPs both for cell proliferation without the application of the magnetic field and under the external constant magnetic field.

## Figures and Tables

**Table 1:** Compositions and selected properties of “blank” gel and ferrogels.

Mark	Content of Mnps in the Reaction Mixture (wt. %)	Equilibrium Swelling Ratio in Water	Content of MNPs in the Gel (wt. %)	Equilibrium Swelling Ratio in 199 Salt Solution
BG	0.0	11.8 ± 0.5	0.00	11.2 ± 0.4
FG1	1.0	12.6 ± 0.6	0.63	11.5 ± 0.5
FG2	2.0	13.2 ± 0.7	1.19	11.3 ± 0.5

**Table 2:** Density of fibroblasts at different experimental conditions. No significant differences were determined.

	Fibroblasts Monolayer Density (cells/cm^2^)		
Experimental Condition	8 Central Wells	16 Peripheral Wells	All 24 Wells
Magnetic field of 400 Oe	5800 ± 500	5700 ± 400	5700 ± 400
Control (absence of magnetic field)	4500 ± 500	4300 ± 500	4400 ± 500

**Table 3:** The effect of a magnetic field on the fibroblasts population number doublings. Data are presented as *X ± m*, n = 54 (9 fields of view per well × 6 wells). A level of significance *p* < 0.05 was determined for FG1 and FG2.

Experimental Condition	BG (2)	FG1 (2)	FG2 (2)	TCPS (2)
Magnetic field of 400 Oe	0.08 ± 0.03	0.16 ± 0.05	0.6 ± 0.1	2.5 ± 0.2
Control (absence of magnetic field)	0.08 ± 0.04	0.35 ± 0.07 *	1.0 ± 0.1 *	2.3 ± 0.2

* significant difference between gel in magnetic field and control.
